# IgA and IgM protein primarily drive plasma corona‐induced adhesion reduction of PLGA nanoparticles in human blood flow

**DOI:** 10.1002/btm2.10064

**Published:** 2017-05-22

**Authors:** Daniel J. Sobczynski, Omolola Eniola‐Adefeso

**Affiliations:** ^1^ Dept. of Chemical Engineering University of Michigan Ann Arbor MI 48109; ^2^ Dept. of Biomedical Engineering University of Michigan Ann Arbor MI 48109; ^3^ Dept. of Macromolecular Science and Engineering University of Michigan Ann Arbor MI 48109

**Keywords:** nanoparticles, plasma protein corona, adhesion efficiency, immunoglobulins, vascular‐targeted carriers

## Abstract

The high abundance of immunoglobulins (Igs) in the plasma protein corona on poly(lactic‐co‐glycolic) acid (PLGA)‐based vascular‐targeted carriers (VTCs) has previously been shown to reduce their adhesion to activated endothelial cells (aECs) in human blood flow. However, the relative role of individual Ig classes (e.g., IgG, IgA, and IgM) in causing adhesion reduction remains largely unknown. Here, we characterized the influence of specific Ig classes in prescribing the binding efficiency of PLGA nano‐sized VTCs in blood flow. Specifically, we evaluated the flow adhesion to aECs of PLGA VTCs with systematic depletion of various Igs in their corona. Adhesion reduction was largely eliminated for PLGA VTCs when all Igs were removed from the corona. Furthermore, re‐addition of IgA or IgM to the Igs‐depleted corona reinstated the low adhesion of PLGA VTCs, as evidenced by ∼40–70% reduction relative to particles with an Igs‐deficient corona. However, re‐addition of a high concentration of IgG to the Igs‐depleted corona did not cause significant adhesion reduction. Overall, the presented results reveal that PLGA VTC adhesion reduction in blood flows is primarily driven by high adsorption of IgA and IgM in the particle corona. Pre‐coating of albumin on PLGA VTCs mitigated the extent of adhesion reduction in plasma for some donors but was largely ineffective in general. Overall, this work may shed light into effective control of protein corona composition, thereby enhancing VTC functionality in vivo for eventual clinical use.

## 1 INTRODUCTION

Vascular‐targeted drug delivery continues to be explored as an efficient, non‐invasive, and promising alternative to current treatment options for a host of diseases, including cancer and coronary artery disease. Vascular‐targeted *nanoparticle* (NP) systems offer the potential to efficiently deliver therapies exclusively to a target site, while also protecting the drug cargo from degradation—resulting in a higher drug efficacy and reduced side effects over current non‐localized, systemic delivery approaches.^1^, ^2^, ^3^, ^4^ However, several challenges still exist for the widespread use of targeted NPs in the clinics, including their inability to effectively navigate the complex vasculature to reach the targeted organ/disease tissue, limited ability to evade the immune system, and the increasing need to tightly control drug release.^5^, ^6^ The rapidly forming *plasma protein corona* on NPs has been implicated in many of the highlighted limitations, specifically in NP clearance, biodistribution, circulation time, targeting, and uptake.^7^, ^8^, ^9^, ^10^

More recently, the plasma protein corona is reported to significantly affect the vascular wall *adhesion efficiency* of polymeric NPs.^5^, ^8^, ^11^, ^12^, ^13^, ^14^ Specifically, NP targeting efficiency to cells or reactive substrates is found to be completely lost (up to 99% reduction) when exposed to plasma or serum, despite a high density of polyethylene glycol coating (PEG) coating on the particle surface.^11^, ^13^, ^14^ These observations were attributed to the significant presence of *immunoglobulins* (Igs) in the corona formed on the NP surface, which prevents adhesive interaction of the targeting ligands with their corresponding cell or surface receptors.^8^, ^11^, ^12^, ^13^, ^14^ However, the precise role of individual Ig protein classes in this process has yet to be studied. Since protein properties vary widely, it is plausible that corona‐derived effects are linked to a single or combination of a few proteins with certain features (e.g., large molecular weight, low off‐rate, and conformational stability) rather than an aggregate effect of the complex corona. For instance, IgG is known to be highly opsonic in nature, and this feature has been linked to its significant presence on NP surface, leading to rapid hepatic uptake and clearance in vivo.^15^, ^16^

This work seeks to evaluate (a) which Ig class(es) in a *vascular‐targeted carrier* (VTC) corona orchestrate their reduced adhesion to vascular wall endothelial cells (ECs) in blood flow and (b) whether a covalent coating of albumin on VTC surface can curtail this adverse effect of Ig protein(s). Specifically, we evaluated the adhesion of poly(lactide‐co‐glycolic) acid (PLGA) particle to activated human ECs in blood flow in which all Igs were eliminated from the PLGA corona relative to the adhesion of particles with IgG, IgM, or IgA individually added (≥70%(m/m)) back into the corona. PLGA VTCs were studied here as our prior work demonstrated the high sensitivity of this material to human plasma and due to its ubiquitous use for construction of many drug delivery systems.^17^ We investigate the individual impact of IgG, IgA, or IgM since these Ig classes collectively constitute the majority (≥98%(m/m)) of plasma Ig content and thus are expected to play significant roles in driving PLGA adhesion reduction. We explored the covalent attachment of albumin to PLGA VTCs given the availability and common use of this dysopsonin protein in increasing circulation time of NPs in the bloodstream by counteracting opsonin (e.g., IgG, complement) adsorption and silencing recognition by the mononuclear phagocytic system (MPS).^7^, ^18^, ^19^, ^20^, ^21^, ^22^ Finally, we focused on non‐PEGylated VTCs to emphasize the effect of the base material (PLGA) in any corona‐derived observations, which is expected to offer critical insight into the amplitude of surface modifications required to abate or alter plasma protein adsorption. Despite important strides made with their use to achieve non‐fouling NPs,^23^ PEG coatings on NPs still cannot completely prevent protein adsorption.^18^ Furthermore, the formation of anti‐PEG antibodies that is expected to accelerate PEG‐NP clearance rate upon re‐administration in vivo remains a major limitation.^24^ Thus, there is a need for more research to elucidate further the mechanism of protein corona‐induced altering of VTCs targeting, which would provide valuable information for the development of novel, NP‐based therapeutics.

## 2 MATERIALS AND METHODS

### 2.1 Particle size and concentration characterization

Carboxylated, 500 nm PLGA particles were obtained from Phosphorex, Inc. (Hopkinton, MA). Carboxylated PLGA particles were dispersed in PBS++ containing 1%(wt/vol) bovine serum albumin (BSA) and then washed with PBS. Particles were incubated in 50 mM MES at pH 7 for ≥ 20 hr prior to DLS measurement of size distribution (Table 1), corresponding to the time required for the NeutrAvidin conjugation.

**Table 1:** PLGA particle size characterization via DLS

**Particle size**	
**Z‐Average (nm)**	**PDI**
440	0.14

### 2.2 Biomolecule surface conjugation

Particles were first conjugated with NeutrAvidin via covalent carbodiimide chemistry followed by linkage to biotinylated sialyl‐Lewis^a^ (sLe^a^) (Glycotech Corporation; Gaithersburg, MD) as described elsewhere.^25^ Briefly, 9.1 × 10^9^ µm^2^/ml particles were incubated with 5 mg/ml NeutrAvidin in MES buffer for 15 min, followed by addition of an equal volume of 75 mg/ml EDAC. For particles with both albumin and NeutrAvidin attached, human serum albumin (Sigma‐Aldrich) was added in a 4:1 ratio with NeutrAvidin during the incubation step. The solution pH was then adjusted to ∼7.4 and placed on an end‐to‐end rotator for ∼20 hr. The conjugation reaction was halted with ∼7.5 mg glycine/ml, and the protein‐coated particles were washed with and re‐suspended in 1 ml of PBS. Attachment of the targeting ligand, sLe^a^, on PLGA particles was performed by suspending NeutrAvidin or NeutrAvidin + Albumin particles in 100 µL of biotinylated sLe^a^ (diluted in PBS++ 1%(wt/vol) BSA) at a concentration of 0.5–3 µg/ml at a surface area to volume ratio of ∼1.5 × 10^9^ µm^2^/ml for 45 min.

### 2.3 Quantification of biomolecule surface density

NeutrAvidin density was quantified using an Attune flow cytometer (Applied Biosystems) via staining conjugated particles with biotin‐PE for ∼20 min, followed by washing with PBS++ 1%(wt/vol) BSA. Quantification of the number of NeutrAvidin and sLe^a^ sites on the particle surface was achieved via use of Quantum R‐PE MESF or FITC calibration beads (Bangs Laboratories) as previously described.^25^ Particle sLe^a^ density (Table 2) was determined via staining with anti‐CLA‐PE (Miltenyi Biotec; San Diego, CA). Rat‐IgM‐PE (Fisher Scientific) was used as the isotype control. Albumin surface density was tested in a similar manner via goat‐anti‐albumin‐FITC (human). Particles stained with goat‐IgG‐FITC at the same concentration served as the isotype control. Table 3 lists the albumin and NeutrAvidin surface densities obtained for PLGA particles in this work.

**Table 2:** PLGA ligand density quantification via flow cytometry

**sLe^**a**^ Ligand Density (#/µm^**2**^)**		
**Particle type**	**Average**	***SEM***
PLGA‐sLe^a^ (Figures 2 and 4)	5,100	1,900
PLGA‐sLe^a^ (Figure 4)	8,500	200
PLGA + Albumin‐sLe^a^ (Figure 4)	8,100	600

**Table 3:** Albumin and NeutrAvidin surface density quantification via flow cytometry

**PLGA Surface Density (#/µm^**2**^)**			
**Particle type**	**Biomolecule**	**Average**	***SEM***
PLGA + Albumin	Albumin	6,300	700
PLGA	NeutrAvidin	1.3e6	N/A
PLGA + Albumin	NeutrAvidin	9.6e4	1.1e4

### 2.4 Human umbilical vein endothelial cell culture

Human umbilical vein endothelial cells (HUVECs) were obtained via a commonly employed collagenase perfusion method.^26^ Umbilical cords were generously donated by the U of M hospital under a Medical School Internal Review Board (IRB‐MED) approved human tissue transfer protocol (HUM00026898). The protocol is exempt from informed consent per federal exemption category #4 of the 45 CFR 46.101.(b). Isolated HUVECs from the umbilical cords were pooled and grown T‐75 tissue culture flasks. Preparation of HUVEC‐coated coverslips for flow chamber assays is described elsewhere.^26^, ^27^ HUVEC monolayers were activated with 1 ng/ml interleukin 1‐beta (IL1‐β) for 4 hr prior to flow experiments to facilitate binding of sLe^a^‐coated particles via interaction with upregulated human E‐selectin.

### 2.5 Preparation of blood and buffer mediums

Human whole blood (WB) was obtained from healthy human donors according to a protocol approved by the University of Michigan Internal Review Board. A written informed consent was obtained from all subjects before blood collection. Acid‐citrate dextrose (ACD) anticoagulant was added at a ratio of 0.14 ml/ml of WB. To obtain plasma, ACD WB was centrifuged at 2,250 g for 20 min at 4°C and again at 6,797 g for 5 min to ensure removal of red and white blood cells and platelets. Plasma was then filtered (0.45 µm pore size) before use.

Depletion of all Igs from plasma was performed using the PureProteome™ Human Albumin/Immunoglobulin Depletion Kit (EMD Millipore). It is expected that this kit induces minimal loss of proteins other than albumin and Ig's, based on the SDS‐PAGE experiments tested by the manufacturer in the user guide document for the product (EMD Millipore Catalog No.: LSKMAGHDKIT). Eluted protein bound to the depletion beads is composed of a concentrated albumin (∼60–70 kDa) and Ig light/heavy chain fraction (30/60 kDa, reduced) bands with minimal non‐specific bands. Specifically, 25 µL of human plasma was diluted with PBS (1:4) to a total volume of 100 µL. This 25% plasma solution was then incubated with 900 µL of the depletion beads for 1 hr at room temperature on an end‐to‐end rotator. The solution was then centrifuged, and the supernatant containing Igs + albumin‐depleted plasma was concentrated using Amicon^®^ Ultra‐4 centrifugal filter units. Albumin was added back to Igs + albumin‐depleted plasma at a concentration of 10 mg/ml to physiological levels of ∼40 mg/ml to obtain Igs‐depleted plasma (contains albumin). A Protein‐A antibody purification kit (Sigma‐Aldrich) was used to obtain IgG‐depleted plasma according to manufacturer protocol. Briefly, 2 ml of plasma was mixed with 4 ml of PBS (plasma + PBS). The plasma + PBS mixture was added to the top of the Protein A cartridge, which binds IgG with high affinity. The filtrate thus contained IgG‐depleted plasma. Column bound IgG was isolated into a separate tube using the elution buffer (low pH glycine solution) and the IgG protein concentrated via Amicon^®^ Ultra‐4 centrifugal filter units. The protein product obtained will be referred to as Iso‐IgG, which contained ∼94%(m/m) IgG as measured via NanoDrop 2000c. IgA was depleted from plasma via a commercially obtained IgA depletion column (GenWay Biotech, San Diego, CA). Native plasma (all Igs present) or IgG‐depleted plasma was diluted with tris‐buffered saline (TBS) (pH = 7.4) to 10%(vol/vol) plasma and 400 µL of this solution incubated with the spin column containing anti‐IgA beads for 15 min. The resulting filtrate contained plasma depleted of IgA only or IgG + IgA. A high concentration IgA1 solution, 70%(m/m), was obtained by first depleting IgG from plasma and then exposing the IgG‐depleted plasma to the IgA depletion column and eluting the column‐bound protein with 0.1 M glycine (pH = 2.5). Commercial IgM (≥95% purity via HPLC) obtained from Sigma‐Aldrich was also used in this study.

### 2.6 Parallel plate flow chamber assay

Flow adhesion assays were performed on a Nikon TE 2000‐S inverted microscope fitted with a digital camera. Circular parallel plate flow chambers (PPFCs) (Glycotech Corporation) containing a straight rectangular channel were used in these assays, as described elsewhere.^11^, ^25^, ^26^, ^27^ Briefly, an activated coverslip containing a monolayer of activated HUVEC was vacuum‐sealed to the bottom of the flow chamber deck. Flow through the chamber was initiated via a programmable syringe pump. Before the flow assays, particles were incubated in plasma in a similar manner as previously described in Sobczynski and Eniola‐Adefeso^12^, with a few key differences. Prior studies with PLGA particles have shown that after ≥5 min of exposure to 100% (i.e., undiluted) plasma, the extent of the adhesion reduction was saturated with no additional reduction observed up to 60 min incubation time.^11^ Since the depletion kits limited the amount of depleted plasma available, incubation of diluted plasma was employed, and particles were incubated for ∼1 hr (Supporting Information Figure S1). The 1 hr incubation time with 25% plasma was chosen here since similar levels of adhesion reduction were achieved with this condition, as compared to ≥5 min 100% plasma incubation,^11^, ^12^ which represents the physiological scenario in vivo. Moreover, a recent study by Tenzer et al.^28^ observed that a protein corona is established within 1 min of plasma exposure and that changes beyond this time, up to 4 hr, occurs only in the amount and not the types of bound proteins.

Particles were incubated in the following media: 25%(vol/vol) native plasma, 25%(vol/vol) IgG‐depleted plasma, 25%(vol/vol) Igs‐depleted plasma (with and without Iso‐IgG, Iso‐IgA, and Purified‐IgM), and PBS. Particles were fluorescently labeled with biotin‐PE and reconstituted in a red blood cells (RBCs)‐in‐viscous buffer (VB; with viscosity matching that of human plasma and consisted of PBS++ 1%(wt/vol) BSA and 1.4%(wt/vol) dextran^13^, ^29^) medium as previously described.^12^ The hematocrit was fixed to 38%(vol/vol) for all experiments. Particles in RBCs‐in‐VB were introduced to the flow chamber for 5 min at a shear rate of 200/s. After the flow experiment, the particle adhesion density (#bound/mm^2^) to HUVEC was obtained by fluorescent imaging along the width of the monolayer at a fixed position from the channel entrance.

### 2.7 SDS‐PAGE

SDS‐PAGE was performed using 4–20% Tris‐Glycine precast gels from Bio‐Rad Laboratories or Life Technologies as previously described in.^12^ For the solutions prepared in the SDS‐PAGE experiments, conditions were diluted to ∼2% plasma solution, and therefore the total protein content during loading is expected to be similar across the different conditions. For the gels characterizing the corona proteins stripped from the NP surface, loading was normalized across the different experimental conditions by fixing the total particle surface area (typically ∼2.54 × 10^8^ µm^2^/ml plasma). Furthermore, the same batch of particles exposed to the same amount of plasma was used on a given day for all conditions to reduce any deviations in expected particle concentration due to counting different particle stocks.

### 2.8 ELISA

Sandwich ELISA was employed for measurement of human plasma IgG, IgA, and IgM concentrations. Clear, 96‐well plates were coated with the appropriate antibody at 1–6 µg/ml overnight (50 µL/well). To capture IgG, IgA, and IgM, goat anti‐human IgG‐unlabeled, Goat anti‐human IgA‐unlabeled, and goat anti‐human IgM‐unlabeled were used as the coating antibodies (Southern Biotech). Plates were washed with PBS + 0.05%(vol/vol) Tween‐20 (wash buffer) and blocked for ∼1 hr with wash buffer containing 10% (vol/vol) FBS (200 µL/well). Plasma was diluted ∼1:1e3–1:1e8 to obtain a linear working range. Quantification was achieved by comparison with a standard curve consisting of commercially obtained IgG, IgA, and IgM solutions ranging from ∼0.3 to 40 ng/ml. For assay detection, horseradish peroxidase (HRP) conjugated antibodies were incubated with samples at ∼0.1–0.8 µg/ml for 1 hr, depending on the specific antibody type. The following detection antibodies (Southern Biotech) at 100 µL/well were used in this assays: mouse anti‐human IgG Fc‐HRP (Clone: H2); mouse anti‐human IgA1‐HRP (Clone: B3506B4); and mouse anti‐human IgM‐HRP (Clone: SA‐DA4). After washing, 50 µL/well of TMB substrate was added, and after ∼5 min, 1N H_2_SO_4_ was added to stop the reaction. Absorbance measurements were taken at 450 nm with 570 nm as the background.

### 2.9 Statistical methods

Data was analyzed using Prism. Data in figures were plotted with standard error and comparisons between adhesion assays were performed using one‐way ANOVA with Tukey post‐test or unpaired *t* test with a confidence interval of 99% (α = .01). Welch's correlation was used for the *t* test if appropriate.

## 3 RESULTS

### 3.1 Assessment of Ig depletion column specificity

**Figure 1:**
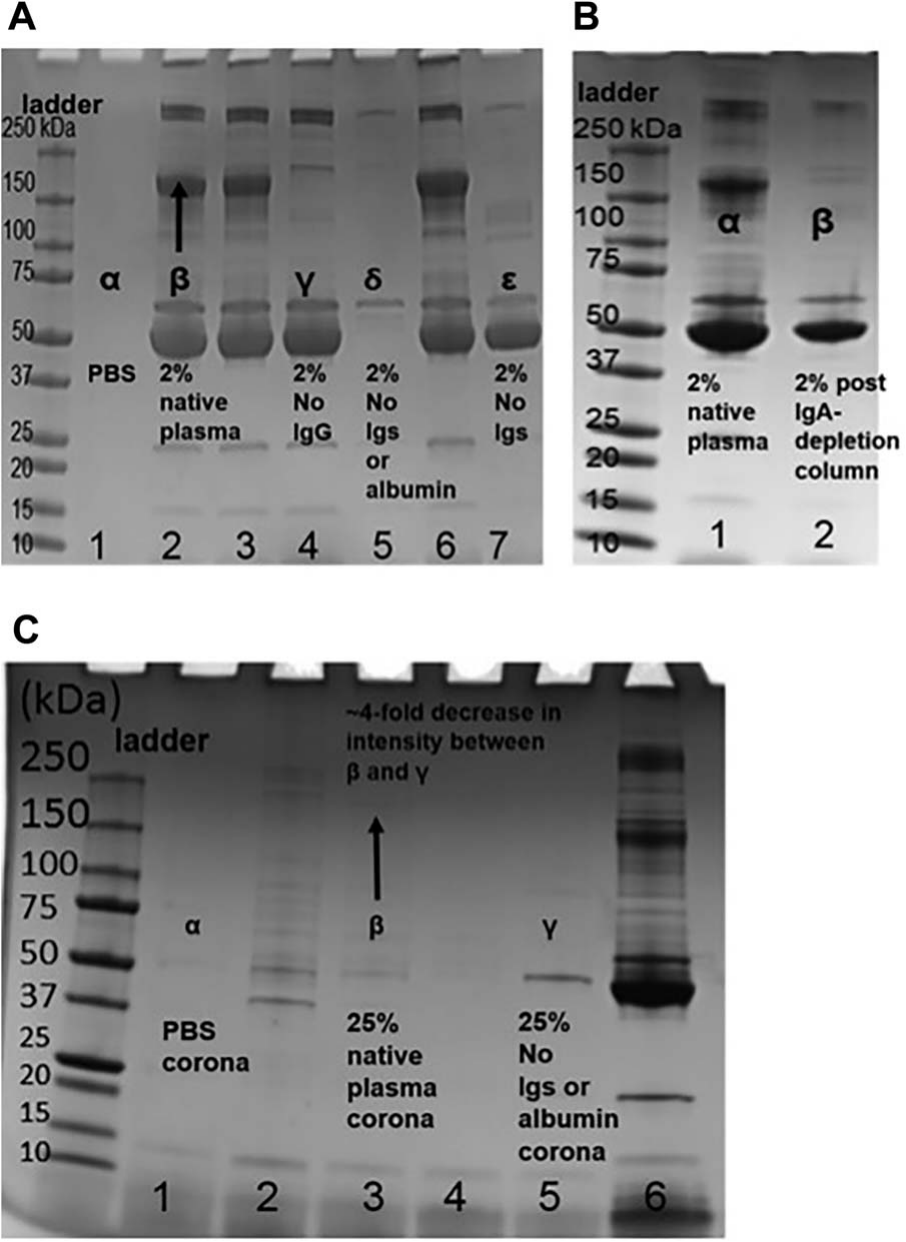
(A) SDS‐PAGE of incubation solutions: Lane 1: (α) PBS, Lane 2: (β) 2% plasma (native), Lane 3: 2% IgG‐depleted plasma post exposure to blocked Protein A column, Lane 4: (γ) 2% plasma (IgG‐depleted), Lane 5: (δ) 2% plasma (Igs + albumin‐depleted), Lane 6: (2% IgG‐depleted plasma + Iso‐IgG), and Lane 7: (ε) 2% plasma (Igs‐depleted). (B) SDS‐PAGE of IgA depleted plasma: Lane 1: (α) 2% plasma (native) and Lane 2: (β) 2% plasma (post exposure to IgA column, “IgA‐depleted”). (C) SDS‐PAGE of PLGA microparticle corona formed from: Lane 1: (α) PBS, Lane 2: 100% plasma, Lane 3: (β) 25% plasma (native) Lane 4: 5% plasma, Lane 5: (γ) 25% plasma (Igs + albumin‐depleted), and Lane 6: no corona, free plasma (native)

It is critical to assess the specificity of the commercially obtained depletion kits employed in this work for the removal of IgG, IgA, and other Igs from human plasma. Both IgG and IgA in blood plasma are ∼150 kDa in size, as confirmed via SDS‐PAGE of commercially obtained solutions (Supporting Information Figure S2). Table 4 lists the % IgG, IgM, and IgA retained in plasma post exposure to the different columns/beads relative to plasma as obtained via sandwich ELISA. The SDS‐PAGE samples in Figure 1 show visual depletion of the ∼150 kDa band for IgG and Igs‐depleted plasma samples (Figure 1A), as well as IgA‐depleted samples (Figure 1B). Overall, the Protein A column designed to deplete only IgG showed relatively high specificity, removing ∼92% of IgG and retaining 80% of IgA relative to native plasma. Some loss in IgM was observed relative to native plasma (∼50%). On the contrary, the IgA depletion column was largely nonspecific as the “IgA‐depleted” sample was also devoid of ∼99% of IgG and IgM relative to native plasma. Similar results were observed for the IgG + IgA‐depleted sample. The Igs‐depleted plasma showed no detectable signal for IgG, IgM, or IgA via ELISA.

**Table 4:** Ig concentration via ELISA post depletion column/bead exposure

**Sample type**	**% IgG retained**	**% IgM retained**	**% IgA1 retained**
IgG depleted	7.9 ± 6.4	54.4 ± 1.6	80.2 ± 1.5
IgA depleted	1.4 ± 0.7	1.0 ± 0.7	0
IgG + IgA depleted	1.4 ± 0.3	0	0
Igs depleted	0	0	0

To confirm that a significant amount of Igs were depleted from the corona when exposed to plasma devoid of Igs, an SDS‐PAGE was performed on the proteins removed from the corona of PLGA particles incubated in buffer, native plasma, and Igs + albumin‐depleted plasma (Figure 1C). An approximate fourfold decrease in the intensity of the ∼150 kDa band (obtained via ImageJ analysis) was observed in the corona of particles exposed to 25% Igs + albumin‐depleted plasma relative to 25% native plasma. Furthermore, the intensity value of the IgG/IgA band obtained from 25% Igs + albumin‐depleted plasma matched closely with that of the corona formed from PBS incubated particles, suggesting a minimal presence of Ig protein in the corona of this sample.

### 3.2 Evaluation of the extent of PLGA adhesion recovery prompted by depletion of all Ig proteins or IgG only from the plasma corona

**Figure 2:**
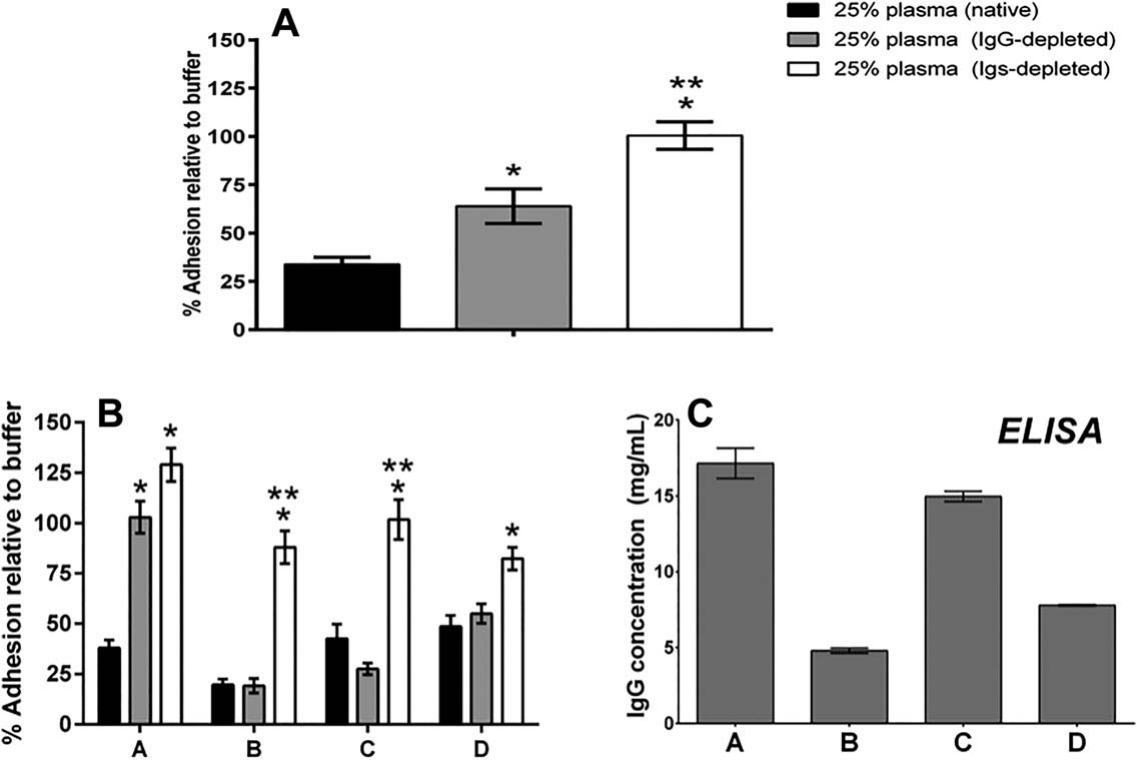
(A) Adhesion of sle^a^‐targeted PLGA particles incubated for 1 hr in 25% plasma (native), 25% plasma (IgG‐depleted), 25% plasma (Igs‐depleted), prior to a parallel plate flow chamber assay in RBCs‐in‐VB (38%(vol/vol) hematocrit) at 200/s. * = *p* < .01 compared to 25% plasma (native) trial, ** = *p* < .01 compared to 25% IgG‐depleted plasma trial, concentration = 1 × 10^6^ # particles/ml, *n* = 4 (averaged across donors A, B, C, and D). (B) PLGA particle adhesion in the same mediums as (a) but on a donor‐to‐donor basis. (C) ELISA of IgG concentration in the various donors

Previous work showed that PLGA microparticles exhibited significant adhesion improvement when exposed to Igs + albumin‐depleted relative to native plasma.^11^ Thus, to establish a baseline with this previous work, the flow adhesion of PLGA NPs incubated in 25% plasma (native) compared to incubation in 25% plasma depleted of all Igs (Igs‐depleted) and suspended in an RBCs‐in‐VB medium was evaluated within the PPFC. All adhesion data obtained were normalized to the adhesion of buffer (PBS)‐incubated NPs perfused through the channel also in the RBCs‐in‐VB medium. Overall, as shown in Figure 2A, the adhesion of PLGA NPs with a corona formed in native plasma was only 34 ± 4% of the adhesion observed for the buffer control (See Supporting Information Figure S3 for representative fluorescent image of particle adhesion). This result is consistent with previous reports that exposure to plasma in whole blood or plasma flow resulted in significant reduction of PLGA particle adhesion to the endothelium. In contrast, no adhesion reduction was observed for PLGA NPs with a corona formed in Ig‐depleted plasma relative to the buffer control. To determine whether the observations of full adhesion recovery with all Igs depletion was consistent across all subjects tested, we examined the impact of donor‐to‐donor variation on particle adhesion with native versus Ig‐depleted plasma. For all donors tested, PLGA NPs with an Ig‐deficient protein corona exhibited major adhesion recovery (≥80%) relative to the buffer control (Figure 2B), confirming that Igs are responsible for the minimal adhesion of PLGA in plasma or plasma containing medium even in the presence of albumin.

We then probed whether IgG, which is abundant in human plasma (∼75%(m/m)), drives the observed PLGA NP adhesion reduction upon plasma exposure by exclusive depletion of this protein from the native plasma. On average, PLGA NPs exposed to IgG‐depleted plasma retained 64 ± 9% of the adhesion observed relative to buffer control; this was significant compared to the relative adhesion observed for particles with a native plasma corona. However, when observing individual donors, a significant recovery in adhesion was only seen for NPs exposed to IgG‐depleted plasma from one donor, donor A, relative to the native plasma from the same donor (Figure 2B). Specifically, there was no significant difference in adhesion of NPs with an IgG‐depleted versus Igs‐depleted corona or buffer control for donor A blood. For the rest of the donors, the levels of particle adhesion with the IgG‐depleted corona were not significantly different from that obtained with the native plasma corona, i.e. the adverse effect of plasma protein persisted, which suggests a secondary role of IgG in conferring the plasma‐induced reduced adhesion of PLGA. Instead, it is likely that the observation of an adhesion recovery for particles exposed to IgG‐depleted donor A plasma is simply an anomaly.

To determine if the unique, dominant role of IgG in conferring the reduced adhesion of PLGA particles in the donor A blood medium is linked to variation in protein concentration in this donor relative to others, the plasma IgG concentration was measured for the different donors evaluated (Figure 2C). Results show that donor A had the highest IgG concentration at ∼17 mg/ml followed closely by donor C at 15 mg/ml, while the other two donors (B, D) had significantly lower levels at ∼5–8 mg/ml. Human plasma IgG concentrations typically range between 7 and 16 mg/ml,^30^ and thus, the high physiological IgG concentration in donor A plasma may in part explain the adhesion recovery for NPs with an IgG‐deficient corona formed from donor A's plasma. However, it is possible that variation in IgG affinity for PLGA across donors also contributes to the observed deviations in particle adhesion between donors in the absence of IgG. As such, SDS‐PAGE was performed for NPs incubated in native plasma from donors A, C, and D. The PLGA corona formed from donor A showed a noticeably heavier adsorption of the ∼150 kDa Ig band (∼12‐fold increase in intensity) when compared head‐to‐head with corona formed from donor C or D plasma (Supporting Information Figure S4). Since donors A and C have a similar plasma level of IgG, the high presence of the ∼150 kDa band in the corona acquired from “A” relative to C plasma suggests that IgG affinity for PLGA also contributes to the significant adhesion recovery observed upon removal of IgG from donor A's plasma.

### 3.3 Evaluation of how adsorption of specific Ig classes to plasma corona affects PLGA NP adhesion

**Figure 3:**
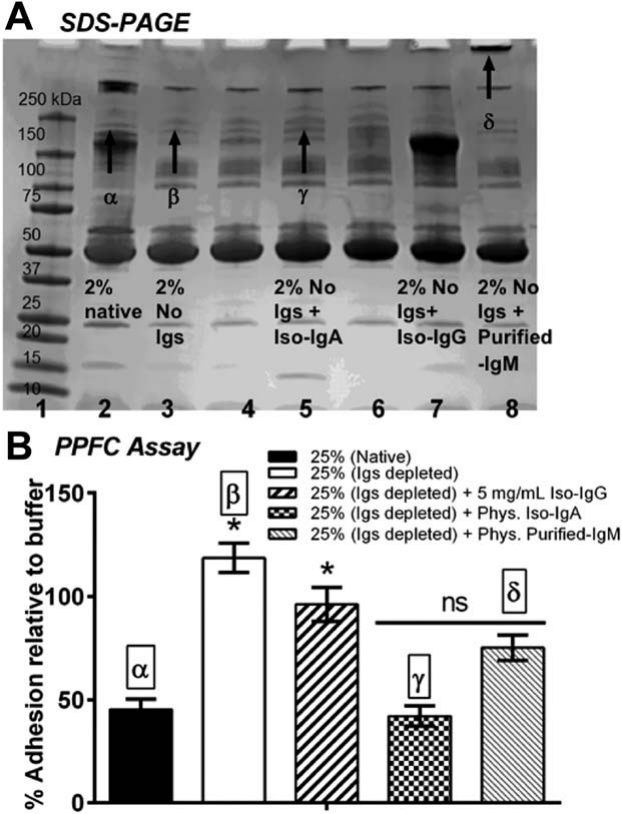
(A) SDS‐PAGE of the 2% plasma solutions used in the flow assay; Lane 1: molecular weight ladder, Lane 2: (α) plasma (native), Lane 3: (β) plasma (Igs‐depleted), Lane 4: plasma (Igs depleted) + 1 mg/ml Iso‐IgA (obtained from native plasma), Lane 5: (γ) plasma (Igs‐depleted) + 0.8 mg/ml Iso‐IgA (obtained from IgG‐depleted plasma), Lane 6: plasma (Igs‐depleted) + IgA (commercial), Lane 7: plasma (Igs‐depleted) + 5 mg/ml Iso‐IgG, Lane 8: (δ) plasma (Igs‐depleted) + 0.8 mg/ml (above physiological level) purified‐IgM. (B) Adhesion of sle^a^‐targeted PLGA particles incubated for 1 hr in 25% plasma (native), 25% plasma (Igs‐depleted), 25% plasma (Igs‐depleted) + 5 mg/ml Iso‐IgG, 0.6–0.8 mg/L Iso‐IgA, and ∼0.2 mg/ml commercial IgM prior to a parallel plate flow chamber assay in RBCs‐in‐VB (38% hematocrit) at 200/s. * = *p* < .01 compared to 25% plasma (native) trial, concentration 1 × 10^6^ # particles/ml

The lack of a full recovery in the absence of IgG, unlike observed with the deletion of all Igs from above, suggests that other Ig types also play a role in the plasma corona‐induced blocking of PLGA NP adhesion. Since it was not possible to exclusively remove the other main Ig types as performed with IgG, we examined the inverse experiment—total removal of Ig proteins followed by a systematic re‐addition of a particular class (IgG, IgA, or IgM) to the PLGA corona. It is important to note here that solely IgA1 was tested in the ELISA experiments since ∼80–90%(m/m) of IgA in blood consists of IgA1 (monomeric structure).^31^ The composition and concentration of the different Ig solutions used in the re‐addition experiments were denoted as follows: “Iso‐IgG” (∼94%(m/m) IgG, ∼5 mg/ml), “Iso‐IgA” (∼70%(m/m) IgA1, ∼0.6–0.8 mg/ml) (See Supporting Information Figure S5) and “Purified‐IgM”. Iso‐IgA and Purified‐IgM were re‐added at physiological levels given their relatively pure composition and to best represent the individual impact of these Ig classes on PLGA adhesion reduction in vivo. Physiological concentrations of IgA1 and IgM were based on the ELISA measurements (Supporting Information Figure S6). Conversely, a high Iso‐IgG concentration (5 mg/ml for 25% plasma solution) was employed in the re‐addition experiment to mimic donor A's plasma conditions, where exclusive removal of IgG from the corona resulted in a major, significant increase in adhesion efficiency. Figure 3A shows SDS‐PAGE of the various protein solutions in which particle coronae were formed.

Figure 3B shows the relative adhesion levels obtained for PLGA NPs incubated in the various solutions characterized in Figure 3A. Overall, the removal of all Igs plus re‐addition of the ∼160 kDa (IgA‐rich) band corresponds to significant adhesion reduction, 35 ± 4%, relative to the removal of all Igs without re‐addition (Supporting Information Figure S3). Furthermore, this level of particle adhesion obtained with re‐addition of IgA was not significant from the level obtained for particles exposed to native plasma (38 ± 4% relative to the removal of all Igs). When the Igs‐depleted NP corona is enriched with Iso‐IgG, adhesion was not significantly different from the adhesion observed for NPs with just an Ig‐depleted corona (*p* = .0915), re‐affirming the minimal role of IgG in causing adhesion deficiency of PLGA particles. When Purified‐IgM was re‐added to the Igs‐depleted corona, NPs again exhibited a slight, but significant, reduction in adhesion level, 64 ± 5%, relative to NPs with just an Igs‐depleted corona. This level of adhesion was, again, not significantly different (*p* = .103) than the adhesion of NPs with a native plasma corona.

### 3.4 Albumin protein attachment to mitigate Ig‐induced PLGA adhesion reduction

**Figure 4:**
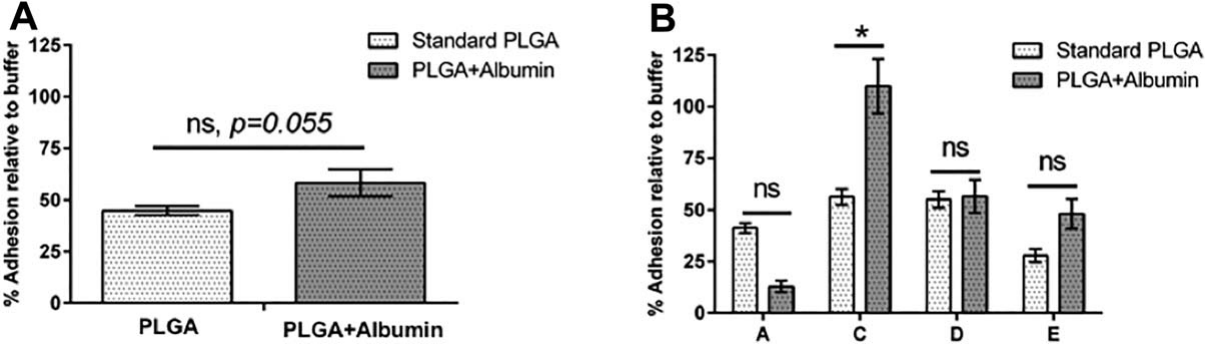
Adhesion of PLGA‐sLe^a^ and PLGA + Albumin‐sLe^a^ particles incubated for 1 hr in 25% plasma (native) averaged from (A) various donors and (B) between individuals relative to RBCs‐in‐VB (38%(vol/vol) hematocrit) at 200/s. * = *p* < .01 compared to PLGA‐sLe^a^ particle trial, concentration 1 × 10^6^ # particles/ml

To determine if the Ig corona‐induced reduced adhesion efficiency of PLGA particles could be neutralized, we evaluated the flow adhesion of albumin‐conjugated PLGA (PLGA + Albumin) particles exposed to native plasma and buffer and compared the results to the adhesion of standard PLGA particles (Figure 4). On average, there was no significant difference in the extent of adhesion (relative to buffer) between standard PLGA‐sLe^a^ and PLGA + Albumin‐sLe^a^ particles (∼8,000 sites/µm^2^) with a native plasma corona when averaged across several donors (Figure 4A). However, analysis of the adhesion of standard and albumin‐conjugated particles between individual donors revealed that the albumin‐conjugated particles performed at 54 ± 13% higher adhesion efficiency relative to that of the standard PLGA when the coronae were derived from donor C's plasma (Figure 4B). Furthermore, an SDS‐PAGE gel analysis of the particle corona upon exposure to donor C plasma shows a ∼twofold decrease in intensity of the ∼150 kDa band (Ig‐rich) band for the corona stripped from the albumin‐conjugated relative to the standard PLGA particles (intensity normalized to respective particle PBS corona; Supporting Information Figure S7). PLGA + Albumin particle adhesion was also slightly increased relative to standard PLGA when exposed to donor E (20 ± 8% increase); however, this was not significant when tested via one‐way ANOVA (*p* = .4620). In the case of particle exposure to donor A and D plasma, no significant improvement in adhesion efficiency was observed between standard and albumin‐conjugated PLGA particles (donor A, *p* = .1930; donor D, *p* > .9999).

## 4 DISCUSSION

Due to improved insight into vascular disease progression as well as NP interaction with blood flow components, vascular targeting remains a viable alternative to invasive surgical procedures and poorly localizing therapies that cause a host of detrimental side effects to patients.^1^, ^3^, ^32^ Recently, the drug carrier's plasma protein corona has been identified to exert an adverse effect on particle adhesion efficiency.^8^, ^11^, ^13^, ^14^ Thus, it is of great interest to the field to determine whether corona‐induced adhesion reduction is driven by specific proteins (e.g., size, surface affinity), as this would offer key insight into the intelligent design of highly efficient VTCs. Moreover, a growing body of literature has focused on “exploiting” the protein corona by attracting a high presence of specific protein types into the corona to improve cell targeting and uptake efficiency.^10^, ^33^, ^34^, ^35^, ^36^

The data presented in this study shows that PLGA adhesion reduction in blood flow is primarily driven by the presence of IgA and IgM in the corona. Removing IgG exclusively from the corona did not result in PLGA adhesion recovery except for particles exposed to donor A plasma; however, significant improvement in adhesion efficiency was observed when all Igs were depleted from the corona. Re‐addition of isolated IgG to the corona had minimal effect in orchestrating adhesion reduction, while re‐addition of IgA or IgM resulted in significant adhesion knockdown relative to particles with all Igs depleted from the corona. To mitigate Ig‐induced adhesion reduction, albumin was pre‐coated onto PLGA particles but only effectively restored the adhesion efficiency compared to normal PLGA particles for 25% of donors tested.

Given the high abundance of IgG in plasma and its well‐known opsonic nature, it was surprising that the removal of IgG from the corona did not significantly restore adhesion efficiency except one donor—A. We hypothesize that PLGA adhesion was effectively restored when the corona was depleted of donor A's IgG due to this donor having a relatively high plasma IgG concentration (Figure 2C) and a seemingly higher affinity for the PLGA corona compared with IgG in other donors (Supporting Information Figure S4). As such, we conclude that donor A may simply be an anomaly, especially as the re‐addition of isolated IgG, at high concentration, to the PLGA corona depleted of all Igs for all donors (including donor A) did not yield improvement in particle adhesion efficiency. It is worth noting that IgG, unlike IgA or IgM, is composed of four different subclasses (IgG1, IgG2, IgG3, and IgG4) that have been reported to exhibit varying levels of uptake and facilitation of macrophage binding and are known to vary in their serum concentration and composition across different individuals.^37^, ^38^, ^39^, ^40^, ^41^ Specifically, IgG3 has been observed to exert the highest bacterial uptake relative to the other subclasses via opsonization as well as facilitate macrophage binding more efficiently than IgG1.^39^, ^42^ Therefore, the observed dominant role of IgG in conferring the reduced adhesion of PLGA NPs in donor A plasma could be linked to a dominance of one IgG subclass versus another in this donor relative to other donors.

The significant adhesion impairment observed upon re‐addition of Purified‐IgM and IgA1 to the corona implicates these proteins as the major driver of PLGA adhesion reduction. IgM has unique features compared to IgG, such as its lower plasma concentration, which might be expected to diminish any potential impact of this protein class on adhesion reduction.^21^ However, IgM is also substantially larger (∼900 kDa) than all other Ig classes (∼150–200 kDa) (Supporting Information Figure S2), which may promote a larger steric interference of receptor‐ligand interactions at the particle surface, thereby weakening adhesion kinetics despite its lower plasma and expected corona concentration than IgG, for instance. While IgA has similar molecular weight to IgG, its dominant role in causing PLGA adhesion reduction could be linked to the similarities of this protein's structural features with IgM, such as the short tailpieces of their CH_3_ domains bonded to J‐chains through disulfide bonds in multimeric forms.^37^ Although this work focused on testing monomeric IgA1 due to its abundance in serum, dimeric IgA (like pentameric IgM) is still present in serum and is much larger than IgG and thus may be more likely to disrupt PLGA targeting efficiency at the NP surface. Also, IgM and IgA also possess similar κ and λ light chains which may contribute to the similar adverse effects on adhesion observed upon adsorption of these Ig classes in the protein corona.^43^ It is important to mention that IgD (∼180 kDa) and IgE (∼200 kDa) may contribute to some of these observations but it is hypothesized that this is unlikely given their extremely low plasma concentration (2–3 orders of magnitude below physiological IgM level).^44^, ^45^ Last, while fibrinogen and fibronectin are also present at high levels and have a strong influence on cellular adhesion, it is unlikely that a non‐specific depletion of these proteins contribute to PLGA particle adhesion recovery with Ig depletion due to the confirmed specificity of the depletion kits use. Moreover, a recent work compared the impact of serum, which is plasma depleted of clotting factors along with fibrinogen and to some extent fibronectin, to plasma and showed the serum acquired corona conferred a stronger adverse effect on PLGA particle adhesion.^12^ Thus, it is unlikely that these proteins contribute to the depletion effects reported in this work.

Given the broad use of PLGA for drug delivery applications and prior FDA approval status, strategies to mitigate the Ig‐induced reduction in targeted adhesion to the vascular endothelium are of interest. While PEGylation of NP surfaces has been extensively explored, and shown to reduce protein adsorption, increase circulation time in vivo, and improve adhesion kinetics in blood flow,^46^, ^47^ it is also widely known that PEGylation does not eliminate protein adsorption.^11^, ^14^, ^18^, ^23^ Furthermore, corona‐induced adhesion reduction of PLGA and other materials has previously been shown to persist in the presence of high surface PEG densities. Attachment of dysopsonin material coatings (e.g., albumin) may serve as a promising avenue to mitigate the extent of corona‐induced adhesion reduction given the utility of this approach in the drug delivery space and recent FDA approval of albumin nanoparticle formulations for cancer treatment (e.g., Abraxane^®^).^15^, ^48^, ^49^, ^50^, ^51^ Specifically, dysopsonin coatings like albumin are known to silence interaction with the MPS system, increase circulation time in vivo, and reduce opsonic protein adsorption—similar to the action of PEG.^7^, ^15^, ^18^, ^19^, ^20^, ^21^, ^22^, ^48^, ^52^ Thus, covalent attachment of albumin to PLGA NPs was explored here as an avenue to abate Ig‐induced adhesion reduction. However, pre‐conjugating PLGA with albumin largely had a minimal effect in restoring PLGA adhesion efficiency upon plasma exposure, except donor C plasma, suggesting that the positive adhesion effect of albumin may be donor‐dependent. Indeed, the corona obtained from donor C native plasma contained a 12‐fold reduction in intensity of the ∼150 kDa band compared to donor A (Supporting Information Figure S4). Thus, it is likely easier for the albumin‐conjugated NPs to resist IgA adsorption in donor C plasma compared to donor A. Overall, given that albumin was only effective for donor C, other avenues for achieving non‐fouling PLGA VTCs still needs to be explored. One possibility may be the use of zwitterionic functionalities on PLGA surfaces, as these materials have shown remarkable promise in eliminating protein adsorption to the particle surface.^53^, ^54^ Though, it remains largely unknown whether these biocompatible coatings will eliminate corona‐induced reduced particle adhesion efficiency to HUVEC in the context of the complex, dynamic blood flow environment.^53^, ^55^, ^56^, ^57^, ^58^

## 5 CONCLUSIONS

This study revealed that IgA and IgM proteins primarily drive adhesion reduction of plasma‐exposed PLGA NPs. These observations will hopefully shed light into the design methodology of high‐efficient VTCs capable of evading corona‐induced adhesion reduction in blood flow. Given previous studies, which have demonstrated the inherent limitations of PEG coatings, it will be of great interest to explore employment of zwitterionic or carbohydrate coatings to achieve high‐efficient binding PLGA NPs. Finally, this study is inherently limited given its use of in vitro assays, which fail to capture the added complexity of the in vivo corona. Indeed, future studies have will transition toward probing differential effects of protein corona fouling between in vitro and in vivo exposed particles.

## Supporting information

Additional Supporting Information may be found online in the supporting information tab for this article.

Supporting Figures
