# Supplementary material for: IgA and IgM protein primarily drive plasma corona‐induced adhesion reduction of PLGA nanoparticles in human blood flow
Source: Bioeng Transl Med. 2017 May 22;2(2):180–90. doi: 10.1002/btm2.10064 (PMC5579729; doi:10.1002/btm2.10064)
Supplement: Supplementary file 1 — Supporting Figures [file BTM2-2-180-s001.docx]

**Appendix. Supplementary Data**

**IgA and IgM protein primarily drive plasma corona-induced adhesion reduction of PLGA nanoparticles in human blood flow**





**Supplementary Figure 1.** Head-to-head comparison of adhesion of sle^a^-targeted PLGA particles incubated for 5 min and 1 hr in 25% plasma (native) prior to a parallel plate flow chamber assay in RBCs-in-VB (38 %(v/v) hematocrit) at 200 s^-1^. * = p <0.01 compared to 25% plasma (native) trial via unpaired t-test.


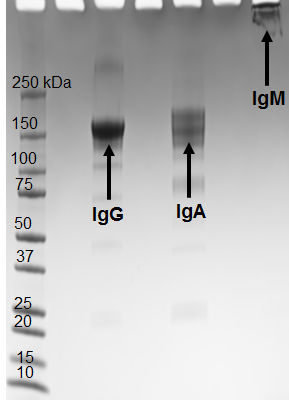


**Supplementary Figure 2.** SDS-PAGE of commercial IgG, IgA, and IgM solutions.


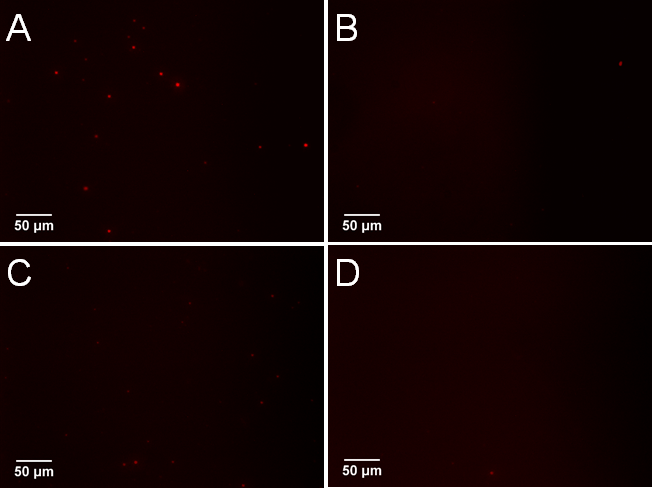


**Supplementary Figure 3.** (A-D) Images of bound PLGA NPs during the PPFC assay experiment for different conditions: (A) PBS corona, (B) 25% plasma corona, (C) No Igs corona, and (D) No Igs + Iso-IgA corona.


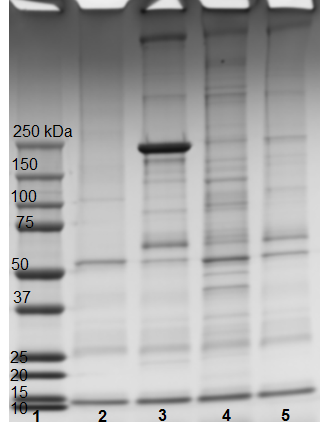


**VB corona**

**100% A corona**

**100% D corona**

**100% C corona**

**Supplementary Figure 4.** SDS-PAGE of PLGA coronae exposed to various 100% donor plasma for 5 min; Lane 1: molecular weight ladder, Lane 2: corona from VB, Lane 3: corona from donor A, Lane 4: corona from donor D, Lane 5: corona from donor C.

**

**

**Supplementary Figure 5:** ELISA Quantification of plasma IgG, IgM, and IgA1 fractions in the eluted product from the Protein A column (Iso-IgG) and where IgG-depleted plasma was exposed to the IgA depletion column (Iso-IgA).

**

**

**Supplementary Figure 6.** ELISA testing of IgA1 and IgM concentrations for different donors.

**
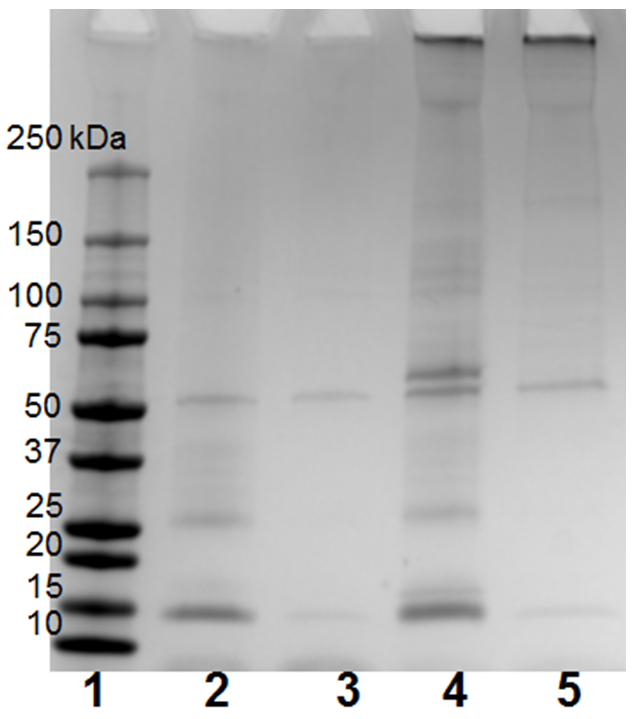
**

**Standard PLGA corona**

**PLGA +albumin corona**

**Control (PBS corona)**

**Supplementary Figure 7.** SDS-PAGE of Standard PLGA-sLe^a^ and PLGA+Albumin-sLe^a^ particle corona post incubation in PBS and 25% plasma (native) from donor C. Lane 1: molecular weight ladder, Lane 2: PBS incubation, Standard PLGA-sLe^a^ Lane 3: PBS incubation, PLGA+Albumin-sLe^a^ Lane 4: 25% plasma (native) incubation, Standard PLGA-sLe^a^ Lane 5: 25% plasma (native) incubation, PLGA+Albumin-sLe^a^.
